# Precision in Medial pivot total knee arthroplasty: A retrospective study on mechanical axis alignment and functional outcomes with CT-guided patient-specific instrumentation

**DOI:** 10.1051/sicotj/2026034

**Published:** 2026-06-24

**Authors:** Fadhil Mat Salleh, Ikram Nizam

**Affiliations:** 1 AOA Accredited Fellow, Mulgrave Private Hospital 48 Blanton Dr Mulgrave VIC 3170 Australia; 2 Ozorthopaedics, Centre for Adult Joint Arthroplasty 1356 High Street Malvern VIC 3144 Australia

**Keywords:** Total knee arthroplasty, Patient-Specific Instrumentation, Mechanical Alignment, Functional outcomes, Medial Pivot TKA

## Abstract

*Background*: Total Knee Arthroplasty (TKA) is the standard treatment for end-stage osteoarthritis, with optimal outcomes dependent on precise mechanical alignment and accurate implant positioning. Patient-specific instrumentation (PSI), facilitated by preoperative CT imaging, has emerged as a technique to enhance surgical precision; however, the relationship between alignment and functional outcomes remains less clear. *Objective*: To evaluate the effectiveness of medial pivot CT-guided PSI TKA in achieving mechanical axis alignment and its correlation with functional outcomes over a 12-month period. *Methods*: This retrospective study analysed 389 primary medial pivot TKA procedures performed by a single surgeon between January 2015 and December 2024 using CT-guided PSI. Functional outcomes were assessed using the Oxford Knee Score (OKS) and Lysholm Knee Score (LKS) preoperatively and at three, six, and twelve months postoperatively. Mechanical alignment was evaluated using the Hip-Knee-Ankle (HKA) angle pre- and postoperatively. Descriptive statistics and paired *t*-tests were applied, with significance set at *p* < 0.05. *Results*: The cohort included 389 patients, 62.7% female, with a mean age of 69.6 years and mean BMI of 31.4 kg/m^2^. PSI significantly improved mechanical alignment, with mean HKA increasing from 172.2° preoperatively to 179.5° postoperatively. Functional outcomes also improved, with mean OKS increasing from 18.5 to 38.6 and mean LKS from 52.4 to 85.5. Patients classified as alignment outliers with severe preoperative deformities showed no significant improvement in alignment but demonstrated substantial functional gains. *Conclusion*: CT-guided PSI improves mechanical alignment and functional outcomes in medial pivot TKA, particularly in mild to moderate deformities. The weak correlation between alignment and functional recovery suggests other factors, including soft tissue balance, prosthesis factors and rehabilitation, to be critical in total knee arthroplasty. Further studies with larger cohorts, longer follow-up, and comparative groups are required.

## Introduction

Total knee arthroplasty (TKA) remains the gold standard for managing end-stage osteoarthritis, providing substantial pain relief and enhanced mobility. The long-term success of TKA, however, relies heavily on achieving precise lower limb alignment and optimal implant positioning, both of which significantly impact prosthesis longevity, functional recovery, and patient satisfaction [1]. Traditional TKA techniques employ standard cutting blocks, which, despite their widespread use, may introduce alignment errors that lead to implant malposition, accelerated wear, and suboptimal functional outcomes [2].

To enhance surgical precision, patient-specific instrumentation (PSI) has emerged as an advanced technique that customizes surgical guides based on individual anatomical differences using preoperative imaging, typically computed tomography (CT) or magnetic resonance imaging (MRI). PSI aims to improve mechanical axis alignment, reduce intraoperative adjustments, and potentially improve postoperative outcomes [3, 4]. Several studies suggest that PSI can reduce the incidence of outliers in mechanical axis alignment, achieving alignment within ±3° of the neutral axis [5]. Additionally, PSI has been shown to offer comparable or slightly superior accuracy in coronal alignment compared to conventional methods [6]. However, while many studies suggest a link between mechanical alignment and functional outcomes, the exact relationship remains debated in the literature.

Given the retrospective nature of this study, causality cannot be conclusively established, and the findings are presented as trends within the study cohort.

Despite these alignment improvements, the relationship between alignment and functional outcomes – measured by validated scoring systems such as the Oxford Knee Score (OKS), Lysholm Knee Score (LKS), and SF-36 – remains ambiguous. While mechanical alignment is an important factor, research has shown that it does not always directly correlate with functional outcomes. Other factors, such as soft tissue balance, rehabilitation, and the surgical technique, also significantly contribute to functional recovery [7–10]. Furthermore, PSI has been associated with reduced surgical time and intraoperative blood loss [9], though these benefits do not always correlate with superior long-term patient satisfaction [10].

Recent technological advancements, including improved imaging modalities, enhanced guide fabrication techniques, and the integration of robotic-assisted surgery, continue to refine PSI’s capabilities [11]. As a result, PSI remains a promising tool in optimizing TKA outcomes, but its role in achieving both mechanical alignment and functional recovery warrants further exploration.

In addition to PSI, the design of the prosthetic knee implant itself plays a critical role in optimizing functional outcomes. One such design, the medial pivot knee, has garnered significant attention in recent years. Medial pivot knees are characterized by an anatomically accurate design, where the femoral component conforms more closely to the medial side of the tibia, aiming to mimic the natural motion of the knee. This design seeks to enhance stability and knee function by promoting natural rolling and sliding during flexion. Previous studies have shown that medial pivot knees tend to offer superior functional outcomes, including improved knee stability and a more natural feeling of movement compared to traditional posterior-stabilized and cruciate-retaining designs [12]. Patients with medial pivot implants have also reported higher satisfaction rates, better knee kinematics, and less anterior knee pain [13]. However, the long-term effects of medial pivot knees are still being explored, and further research is needed to confirm their durability and potential advantages.

### Hypothesis

This study hypothesizes that CT-guided PSI in TKA will improve mechanical axis alignment, and that this improvement may be associated with better functional outcomes, as measured by the Oxford Knee Score (OKS) and Lysholm Knee Score (LKS). However, the exact relationship between alignment and functional recovery remains debated, and this study aims to clarify the role of PSI in this context.

This study aims to evaluate the accuracy of CT-guided PSI in achieving post-operative mechanical axis alignment and its correlation with functional outcomes over a one-year retrospective period. By systematically analysing radiographic alignment and patient-reported outcomes, this research seeks to contribute to the existing literature by providing insights into the role of PSI in modern TKA and assessing its potential to improve short-term functional recovery.

## Materials and methods

### Study design and methodology

This retrospective study analysed primary medial pivot Total Knee Arthroplasty (TKA) procedures performed by a single surgeon between January 2015 and December 2024. Given the retrospective nature of the study, no causal conclusions can be drawn, and the results should be interpreted as trends within the study cohort. The study was approved by the institutional ethics committee and adhered to ethical guidelines for retrospective studies involving human subjects.

**Figure 1:**
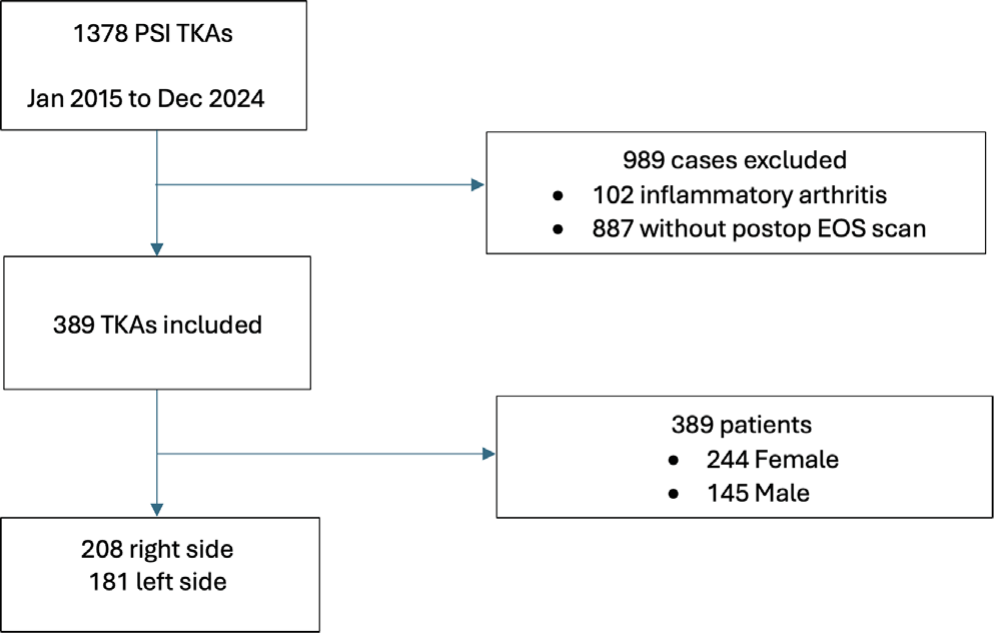
Flowchart.

Inclusion criteria comprised patients who underwent elective primary TKA for primary osteoarthritis and who received surgery using medial pivot CT-guided Patient-Specific Instrumentation (PSI) TKA. Exclusion criteria included patients who underwent conventional TKA, revision TKA, incomplete documentation of functional scores, unavailability of postoperative EOS Imaging scans, or those diagnosed with inflammatory arthritis (Figure 1).

Data were collected using Genie Software Version 9, which recorded demographic information and functional outcome measures. The primary outcome measures included the Oxford Knee Score (OKS) and Lysholm Knee Score (LKS), which were used to evaluate functional recovery. These scores were evaluated preoperatively and postoperatively at 3 months, 6 months, and 1 year.

**Figure 2:**
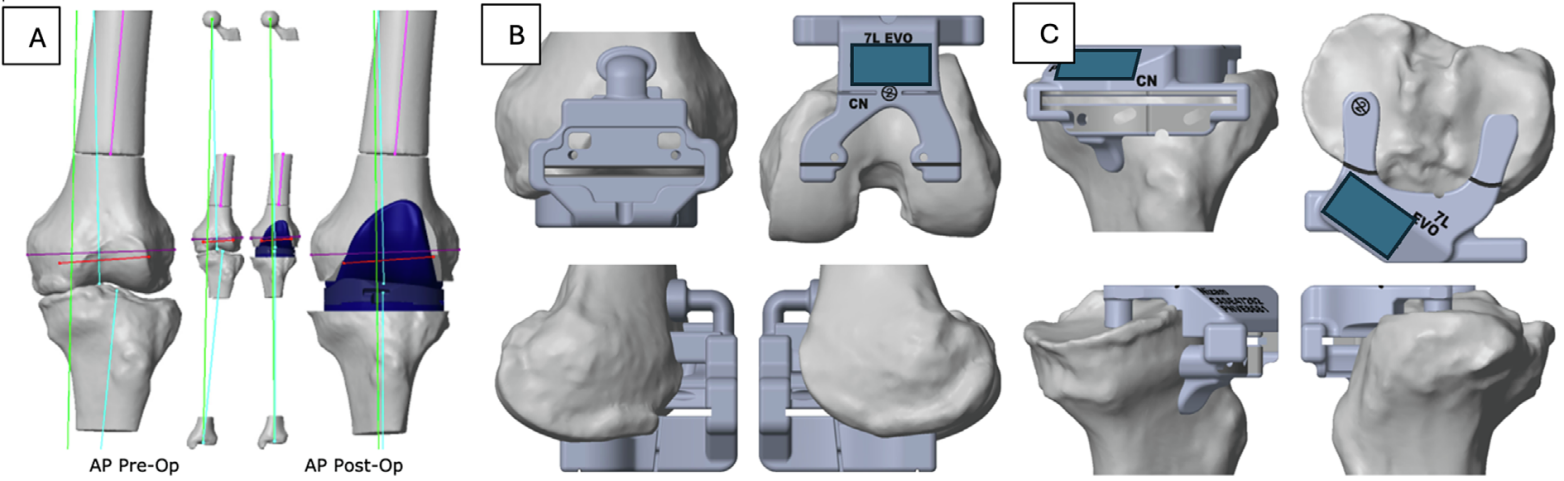
Preoperative planning using Prophecy Evolution Protocol. (A) Pre and postoperative HKA. (B) Femoral guide placement. (C) Tibial guide placement.

Preoperative CT scans were performed using the Prophecy Evolution protocol, which utilized rapid prototyping, computer-assisted design (CAD), and computer-assisted manufacturing (CAM) technologies to create 3D patient-specific cutting blocks (Figure 2). The preliminary surgical plan, including resection levels, alignment, and component sizing, was reviewed and approved by the senior surgeon before the blocks were fabricated by PROPHECY^®^ (Arlington, TN, USA). The preoperative Hip-Knee-Ankle (HKA) angle was determined based on the Prophecy protocol.

The study did not include a control group (e.g., conventional TKA), which limits the ability to directly compare the effectiveness of PSI and medial pivot implants against traditional techniques. The follow-up period for this study was one year, which is a limitation when evaluating long-term outcomes. Further prospective studies with control groups and longer follow-up periods are needed to more thoroughly assess the benefits and limitations of PSI in TKA.

### Surgical technique

All procedures were performed using a standard midvastus approach. The Evolution Medial Pivot Patient-Specific TKA system (Wright Medical, Surgical Specialities Pty Ltd) was used in all patients. The PSI cutting block was placed on the distal femur and proximal tibia according to the preoperative surgical guide. Osteophytes were not removed beforehand to determine the appropriate bony cuts. The thickness of the distal femur, posterior condyle, and proximal tibia cuts was measured and confirmed to match the preoperative planning established by the Prophecy Evolution protocol [14].

Both femoral and tibial components were cemented. Patella resurfacing was selectively performed based on intraoperative findings of the patellofemoral joint (PFJ). Trial implants were used to assess component sizing, ligament stability, range of motion, and patella tracking. The appropriate polyethylene thickness was determined based on intraoperative stability. All patients received local infiltration analgesia as part of their postoperative pain management protocol [15]. Patients were mobilized on the first postoperative day.

The procedures were performed by a single surgeon, which introduces potential selection bias and may limit the generalizability of the findings. Additionally, the study did not include a control group (e.g., conventional TKA), limiting the ability to directly compare the outcomes of PSI and medial pivot implants with traditional techniques.

**Figure 3:**
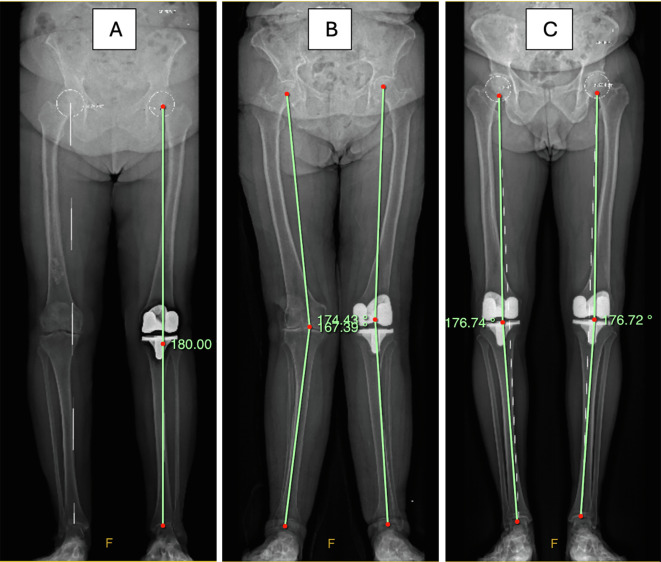
Postoperative EOS scan. (A) Neutral alignment. (B) Valgus alignment (185.57°) in constitutional valgus knee. (C) Bilateral varus alignment (176°) in constitutional varus knee.

Postoperatively, an EOS^TM^ Imaging scan of the hip, knee, and ankle was performed at six months to assess the postoperative HKA (Figure 3). The EOS scan involved full anteroposterior (AP) lower limb standing radiographs, with the patella oriented directly forward. The HKA was defined as the angle between the mechanical axes of the femur and tibia. Deviations from the neutral mechanical axis (180°) were recorded, with an ideal range of ±3°. A mechanical axis less than 180° indicated varus alignment, and more than 180° indicated valgus alignment [16]. Outlier analysis was conducted to determine the proportion of cases exceeding the ±3° deviation.

The postoperative evaluation included EOS scans at six months, but the study’s follow-up period was limited to one year. Further studies with longer follow-up durations are needed to assess the long-term effects of PSI on functional outcomes and implant survivorship.

### Statistical analysis

Statistical analyses were performed using SPSS version 30 (IBM Corp., Armonk, NY). Normality of data distribution was assessed using the Shapiro-Wilk test, and homogeneity of variances was evaluated using Levene’s test. Paired *t*-tests were employed to compare preoperative and postoperative functional outcomes, including the OKS and LKS, as well as mechanical alignment (HKA). A *p*-value of <0.05 was considered statistically significant. Post hoc Bonferroni corrections were applied where applicable.

Descriptive statistics, including mean, standard deviation, and the percentage of cases within ±3° of neutral alignment, were calculated. Outlier analysis was conducted to compare patients within the ±3° alignment range and those outside it. Paired *t*-tests were used to compare pre- and post-operative OKS, LKS, and HKA for both groups.

Multivariate regression models were employed to assess the impact of potential confounding variables, such as age, body mass index (BMI), and preoperative deformity, on functional outcomes and alignment accuracy. However, the results of these models are not fully reported here and should be explored in future studies.

Correlation analysis was performed to examine the relationship between functional outcomes (OKS and LKS) and mechanical alignment (HKA) and to determine the effect of alignment deviations on patient-reported outcomes.

### Limitations

The small sample size of the outlier group (22 patients) limits the statistical power of subgroup analyses and should be considered when interpreting the results. A power analysis was not performed in this study, which limits the ability to assess the statistical significance of smaller effect sizes. Future studies should consider incorporating power analysis to enhance the reliability of the findings. This study also did not analyze potential confounders such as comorbidities, rehabilitation protocols, or implant size, which may affect functional outcomes. Future studies should include these variables to better assess their impact on TKA outcomes.

## Results

A total of 389 patients, predominantly female (*n* = 244, 62.7%), who underwent primary medial pivot Total Knee Arthroplasty (TKA) with CT-guided Patient-Specific Instrumentation (PSI) were included in the study. The study cohort had a mean age of 69.6 years, with a mean body mass index (BMI) of 31.4 kg/m^2^. Preoperative assessments revealed a mean Oxford Knee Score (OKS) of 18.5 and a mean Lysholm Knee Score (LKS) of 52.4. Postoperatively, there was a notable improvement, with the mean OKS rising to 38.6 and the mean LKS increasing to 85.5. With respect to mechanical alignment, the mean preoperative Hip-Knee-Ankle (HKA) angle was 172.2°, which improved to a mean postoperative value of 179.5°. Twenty-two patients were classified as outliers based on post-operative mechanical axis deviations exceeding ±3° from the neutral alignment, while the remaining 367 patients were categorized as non-outliers. The analysis focused on comparing pre- and post-operative functional outcomes (Oxford Knee Score [OKS] and Lysholm Knee Score [LKS]) and mechanical alignment (Hip-Knee-Ankle [HKA] angle) for both groups.

### Paired *t*-test results

The paired *t*-test analysis (Table 1) revealed significant improvements in functional outcomes (OKS and LKS) for both non-outliers and outliers, with both groups showing substantial recovery. However, mechanical alignment (HKA) improved significantly for the non-outliers but showed no significant improvement in the outliers.

- Non-outliers: Significant improvement in OKS, LKS, and HKA post-operatively (all *p*-values <0.0001).
- Outliers: Significant improvement in OKS and LKS, but no significant change in HKA alignment (*p* = 0.215).

**Table 1:** Paired *t*-test results for OKS, LKS, and HKA pre- and post-operative comparisons.

Outcome	Non-outliers (*n* = 367)	Outliers (*n* = 22)
OKS	*t* = −88.57, *p* < 0.0001	*t* = −25.68, *p* < 0.0001
LKS	*t* = −62.74, *p* < 0.0001	*t* = −18.84, *p* < 0.0001
HKA	*t* = −54.79, *p* < 0.0001	*t* = −1.27, *p* = 0.215

### Descriptive statistics

Descriptive statistics (Table 2) of functional outcomes (OKS and LKS) and mechanical alignment (HKA) show that:

- Non-outliers had an average post-operative OKS of 39.87 (±8.3) and an average post-operative LKS of 81.03(±8.9). Their post-operative HKA averaged 179.82° (±3.7), falling within the ideal range of ±3° from neutral alignment.
- Outliers, despite having significant functional improvements, had a lower average post-operative HKA of 175.47° (±5.9), indicating misalignment.

**Table 2:** Descriptive statistics for functional outcomes and mechanical alignment.

Group	Pre-Op OKS	Post-Op OKS	Pre-Op LKS	Post-Op LKS	Pre-Op HKA	Post-Op HKA
Non-Outliers	17.8 (±5.4)	39.9 (±8.3)	44.9 (±6.1)	81.0 (±8.9)	174.9 (±6.2)	179.8 (±3.7)
Outliers	18.7 (±5.7)	36.8 (±6.9)	45.1 (±5.6)	75.3 (±10.2)	172.2 (±7.4)	175.5 (±5.9)

#### Correlation analysis

The correlation analysis (Table 3) between post-operative HKA and functional outcomes (OKS and LKS) showed:

- Non-outliers: Weak to negligible correlations between post-operative HKA and OKS (*r* = 0.0554) and LKS (*r* = −0.0379), indicating that while mechanical alignment improved, it did not strongly correlate with functional recovery.
- Outliers: Also exhibited weak correlations between post-operative HKA and OKS (*r* = 0.0246) and LKS (*r* = −0.0289), reinforcing the idea that alignment may not be the sole contributor to functional recovery in patients with more severe pre-operative deformities.

**Table 3:** Correlation coefficients between HKA and functional outcomes.

Variable	Non-outliers (*n* = 367)	Outliers (*n* = 22)
Pre-Op HKA vs. Post-Op OKS	−0.0287	−0.0397
Post-Op HKA vs. Post-Op OKS	0.0554	0.0246
Pre-Op HKA vs. Post-Op LKS	0.0755	0.1214
Post-Op HKA vs. Post-Op LKS	−0.0379	−0.0289

## Limitations

The small sample size of the outlier group (22 patients) limits the statistical power of subgroup analyses, which should be considered when interpreting these results. A power analysis was not conducted for this study, which limits the ability to assess the statistical significance of smaller effect sizes. Future studies should include a power analysis to improve the robustness of statistical inferences. This study also did not account for potential confounders such as comorbidities, rehabilitation protocols, or implant size, which could influence both functional outcomes and alignment. These variables should be considered in future research.

## Discussion

This study aimed to evaluate the effectiveness of CT-guided Patient-Specific Instrumentation (PSI) in achieving optimal mechanical axis alignment and improving functional outcomes in medial pivot total knee arthroplasty (TKA). The results support the hypothesis that CT-guided PSI significantly improves mechanical alignment, as evidenced by the substantial improvement in the Hip-Knee-Ankle (HKA) angle in the non-outlier group. However, for patients categorized as outliers, PSI demonstrated limited efficacy in achieving neutral mechanical alignment, likely due to severe preoperative deformities such as constitutional varus or valgus. Despite these alignment discrepancies, functional outcomes, as measured by the Oxford Knee Score (OKS) and Lysholm Knee Score (LKS), significantly improved in both the non-outlier and outlier groups, suggesting that PSI may contribute to functional recovery, even when alignment is not fully optimized. However, it is important to acknowledge that the absence of a control group limits the ability to draw definitive conclusions about PSI’s effectiveness in comparison to conventional TKA.

While the improvement in alignment was significant for most patients, the weak correlation between post-operative alignment (HKA) and functional outcomes (OKS and LKS) suggests that mechanical alignment alone may not be the sole determinant of functional recovery [17, 18]. Other factors, such as soft tissue balance, patient rehabilitation, and the surgeon’s technique, likely contribute to these outcomes [19–21]. This finding suggests that while alignment is an important factor, achieving ideal mechanical alignment may not always translate into better functional outcomes, especially in patients with more complex deformities or challenges with soft tissue balancing.

Interestingly, the observed improvement in functional outcomes in the outlier group – despite suboptimal alignment – may also be attributed to the function of medial pivot TKA. Medial pivot knees are designed to replicate the natural kinematics of the knee, providing a highly congruent medial compartment and a less conforming lateral compartment. This design facilitates more natural femoral rollback during flexion, which enhances knee stability and function. As a result, medial pivot designs may be more forgiving of alignment discrepancies, potentially compensating for mechanical misalignments, especially in patients with severe deformities [22]. Studies have shown that medial pivot TKA can yield favourable functional outcomes even in patients with advanced deformities. For example, a study of patients with end-stage arthritis demonstrated significant improvements in the Knee Society Score (KSS) and Oxford Knee Score (OKS), despite the presence of severe deformities. This suggests that the stability and natural kinematics of medial pivot TKA contribute to positive outcomes, even when mechanical alignment is not ideal [23].

Furthermore, a systematic review of long-term outcomes of medial pivot TKA reported a high implant survivorship rate of 98.2% over an average follow-up of 12.4 years. The review also noted significant improvements in patient-reported outcome measures (PROMs), reinforcing the idea that medial pivot TKA can provide durable functional benefits, even in challenging cases with alignment deviations [24]. These findings suggest that the design characteristics of medial pivot TKA may offer enhanced stability and function, mitigating the impact of mechanical alignment deviations on postoperative outcomes.

The results also align with previous studies that suggest PSI can lead to more precise alignment in patients with mild to moderate deformities but is less effective in patients with severe constitutional malalignments [25–28]. However, the small sample size of the outlier group (22 patients) limits the statistical power, and further studies with larger cohorts are needed to provide more robust data on PSI’s role in patients with severe deformities. Future studies should explore how patient-specific factors, such as preoperative deformities and soft tissue conditions, interact with PSI and affect both mechanical alignment and functional recovery. These considerations are vital for refining the use of PSI and improving its effectiveness across diverse patient populations.

### Study limitations

This study is not without its limitations. Firstly, the retrospective design and lack of a control group limit the ability to establish causality between alignment and functional outcomes, as the study does not control for other potential influencing factors, such as rehabilitation protocols or post-operative care strategies. Future prospective randomized control studies would be beneficial in better understanding the causal relationship between mechanical alignment and long-term functional recovery.

Secondly, the relatively small sample size of outlier patients (*n* = 22) reduces the statistical power to detect subtle differences between this group and non-outliers. A larger cohort of outlier patients would provide more robust data on how PSI performs in patients with severe deformities, further clarifying its potential role and limitations in these cases.

Additionally, while the study examined preoperative mechanical alignment, it did not directly assess or account for constitutional varus or valgus, which could have influenced the mechanical alignment and functional recovery of patients. Future studies should incorporate these variables to better understand their impact on PSI outcomes. Moreover, post-operative rehabilitation was not standardized or accounted for, and its variability may have influenced the functional recovery observed in both groups [29].

Lastly, the 1-year follow-up duration in this study may be insufficient to assess the long-term effects of PSI on prosthesis survival and long-term functional outcomes. A longer follow-up period would provide a more comprehensive understanding of the durability and long-term effectiveness of PSI.

## Conclusion

In conclusion, CT-guided Patient-Specific Instrumentation (PSI) has demonstrated significant improvements in mechanical axis alignment and functional outcomes in medial pivot total knee arthroplasty (TKA), particularly in patients without severe preoperative deformities. While PSI effectively enhanced alignment in most patients, its efficacy in patients with severe constitutional varus or valgus deformities was limited, highlighting the importance of individualized surgical planning. However, the retrospective design and lack of a control group limit the ability to make definitive conclusions about PSI’s superiority over conventional techniques.

Although PSI improves mechanical alignment, the weak correlation between alignment and functional recovery observed in this study suggests that other factors, such as soft tissue balance and rehabilitation, are integral to achieving optimal functional outcomes. These findings emphasize the need for a multifaceted approach to TKA, wherein PSI is combined with careful attention to patient-specific factors and rehabilitation strategies.

Future research should focus on expanding the sample size of patients with severe deformities to better understand PSI’s role in these cases. Additionally, incorporating the evaluation of constitutional alignment, soft tissue balancing, and standardized rehabilitation protocols will provide deeper insights into optimizing TKA outcomes. Longer follow-up periods will also be necessary to assess the long-term effects of PSI on functional recovery and implant survivorship. This study underscores the importance of personalized approaches in medial pivot TKA and highlights PSI’s potential to improve alignment and functional recovery, though its full impact is likely influenced by a variety of patient-specific and procedural factors.

## Data Availability

Data and materials are available on request.
